# Enhanced synaptic long-term potentiation in the anterior cingulate cortex of adult wild mice as compared with that in laboratory mice

**DOI:** 10.1186/1756-6606-2-11

**Published:** 2009-05-16

**Authors:** Ming-Gao Zhao, Hiroki Toyoda, Yu-Kun Wang, Min Zhuo

**Affiliations:** 1 School of Pharmacy, Fourth Military Medical University, 17 Changle West Road, Xi'an 710032, Shaanxi, PR China; 2 Department of Physiology, Faculty of Medicine, University of Toronto, 1 King's College Circle, Toronto, M5S 1A8, Canada

## Abstract

Activation of N-methyl D-aspartate (NMDA) receptor is important for learning, memory and persistent pain. Genetic enhancement of NMDA receptor function by overexpressing NR2B subunit significantly enhances hippocampal long-term potentiation (LTP), behavioral learning as well as persistent pain. Recent studies found that NMDA NR2B subunits can undergo long-term upregulation in the brain under certain conditions including peripheral injury and environmental enrichment. Considering the fact that laboratory grown animals live in an artificial comfort environment, we wondered if NMDA receptor functions and its related LTP would differ in animals living in a natural wild environment. In this report we performed whole-cell patch-clamp recordings from both laboratory wild-type mice and wild mice from a natural environment. We found that LTP was significantly enhanced in the anterior cingulate cortex (ACC) of the wild mice as compared with that of laboratory mice. In parallel, NMDA receptor NR2B/total NMDA receptor mediated EPSC ratio was significantly increased in slices of wild mice. Our findings provide the first evidence that NMDA NR2B receptors play an important role in experience-dependent synaptic potentiation within the ACC in wild mice as previously reported in laboratory mice.

## Introduction

The NMDA receptor plays a critical role in synaptic plasticity in many brain regions including the hippocampus, amygdala and anterior cingulate cortex (ACC) [1]. In most central synapses, NMDA receptors are composed of NR1, NR2 (A, B, C, and D), and NR3 (A and B) subunits. The formation of functional NMDA receptors requires a combination of NR1 and at least one NR2 subunit [2]. It is known that the NR2A and NR2B subunits predominate in the forebrain neurons, and the NR2A/NR2B subunit composition determines the functional properties of NMDA receptors [3,4]. Moreover, NMDA receptor subunits can undergo plastic changes in different regions of the brain during early development and different physiological/pathological conditions [2,5-8]. For example, enriched animals display better leaning, enhanced hippocampal LTP, increased NMDA receptor NR2B subunit mediated currents in the forebrain [9,10].

The importance of NMDA receptor NR2B subunit in hippocampal LTP and behavioral learning has been demonstrated by studies using transgenic mice with forebrain overexpression of NR2B subunits [11]. In these transgenic mice, hippocampal LTP is significantly enhanced, along with enhanced learning ability [11] and persistent pain [12]. In the ACC, NMDA receptor-dependent plasticity including LTP and long-term depression, depend on both NR2B and NR2A subunit-containing NMDA receptors [13,14]. NMDA NR2B receptors contribute to LTP induced by different induction protocols in the ACC [14-16]. Our previous study provides strong evidence that NR2B-containing NMDA receptors in the ACC can contribute to the formation of classical contextual fear memory [2,14].

It is well known that experience-dependent neuroanatomical and synaptic plasticity occurs in the brain. Previous studies reported that animals exposure to enriched environments results in increased cognitive and behavioral performances [17-19]. Furthermore, it has also been reported that environmental enrichment delayed the onset of neurodegenerative disorders [20,21], enhanced neurogenesis [22-24] and facilitated LTP [9]. The modification of synaptic plasticity and learning-related behaviors by the environment supports the notion that cognition is constantly influenced by natural selection and survival risks [25,26]. Most of the previous results have been reported in the hippocampus, a brain region thought to be important for spatial memory. However, less information is available for the ACC, a key structure of the forebrain region. The ACC plays an important role in sensory perception (including pain), learning, memory, emotion and executive functions [27]. Using animal models of inflammation or nerve injury, it has been reported that peripheral inflammation/nerve injury caused the long-term enhancement of presynaptic glutamate release and postsynaptic AMPA receptor mediated responses [2,28-30]. In addition, postsynaptic upregulation of NMDA receptor NR2B subunits in the ACC pyramidal neurons has also been reported after tissue inflammation [17]. Thus, it is conceivable that ACC synaptic functions may be modified by the natural environment. In this study, we took a different approach from previous studies of laboratory mice in enriched environment. We performed electrophysiological recordings from brain slices of wild mice obtained in a large city environment. We predict that these wild mice may have enhanced synaptic functions in the ACC, considering that they need to perform extra efforts daily to seek food and avoid dangerous predators.

## Results

**Figure 1:**
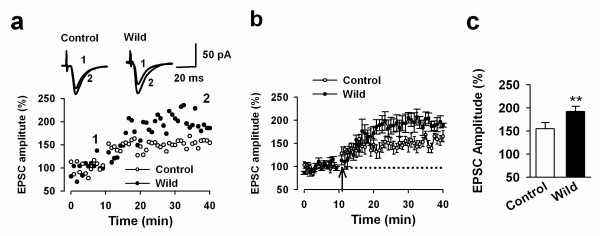
**Enhanced LTP induced by the pairing protocol in the ACC of wild mice**. ***a***: Examples of synaptic potentiation of EPSCs in the layer II/III pyramidal cells of a wild mouse (filled circles) as compared with that in a laboratory (control) mouse (open circles); Inset: sample traces show averages of EPSCs 5 min before (1) and 30 min after (2) the paired training; ***b***: Summarized data for LTP in slices of wild mice (filled circles) and control mice (open circles); ***c***: Summarized data of EPSC amplitude 30 min after LTP induction in slices of control and wild mice. **P < 0.01, compared with laboratory group. Data are presented as mean ± s.e.m.

In our previous studies, we reported that laboratory mice exposed to an enriched environment (EE) showed enhanced long-term plasticity in the ACC [10]. Considering wild mice have developed in a sophisticated city environment, we expect that LTP may be enhanced in the ACC of the wild mice as compared with laboratory mice. We performed whole-cell patch-clamp recordings in visually identified pyramidal neurons in layer II/III of ACC slices. The pyramidal cells are further confirmed by the typical firing pattern induced by postsynaptic injection of depolarized currents. As previously reported [14], the pairing induction protocol produced a significant, long-lasting potentiation of synaptic responses in ACC slices of the control mice. In ACC slices of wild mice, we did not observe any obvious morphological differences. Furthermore, basic synaptic responses evoked by focal electrical stimulation are similar between slices of wild mice and that of control mice. We found, however, that the pairing protocol induced great LTP in slices of wild mice (see Figure 1). Interestingly, a comparison between control and wild mice revealed a significant increase in LTP in slices of wild mice (control group: 154.9 ± 13.9%, n = 6 slices from 4 mice; wild group: 192.6 ± 10.9%, n = 9 from 4 mice; *t*-test, *P *< 0.01; Fig. 1a–c). This suggests that natural environment affects synaptic potentiation in the ACC.

**Figure 2:**
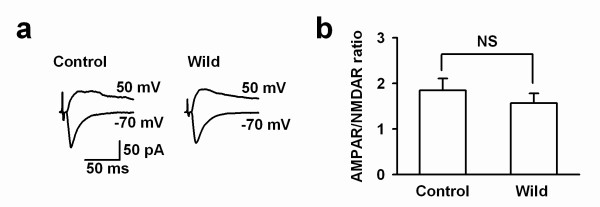
**AMPA/NMDA ratio in the ACC of wild mice**. ***a***: Whole-cell recordings in pyramidal neurons from a laboratory and a wild mouse. AMPA and NMDA EPSCs were recorded at -70 mV and + 40 mV respectively. ***b***: Summary of the AMPA/NMDA ratio in neurons from laboratory and wild mice.

One possible mechanism of enhanced LTP in the wild mice is increased total NMDA current in ACC synapses. To test this, AMPA receptor mediated EPSC/NMDA receptor mediated EPSC ratio was calculated from the peak amplitude of the CNQX-sensitive component measured at -70 mV and the peak amplitude of CNQX-resistant component measured at +40 mV. Analysis of the currents obtained from the laboratory and wild mice showed that there was no difference in the AMPA/NMDA ratio (laboratory group: 1.85 ± 0.26, n = 5 slices from 4 mice; wild group: 1.57 ± 0.27, n = 9 from 4 mice; *t*-test, *P *> 0.05; Fig. 2a and 2b).

**Figure 3:**
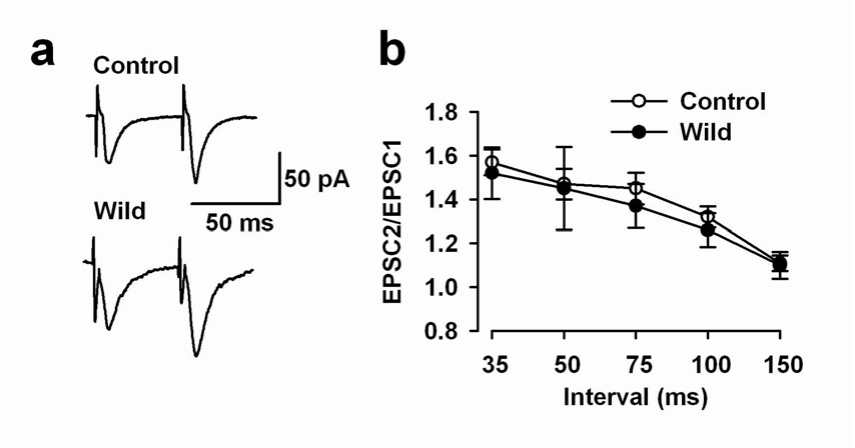
**Paired-pulse facilitation (PPF) is similar in the ACC of wild and laboratory mice**. ***a***: Examples of paired-pulse traces at an interval of 50 ms in wild and control mice. Recordings were performed from layer II/III pyramidal cells. ***b***: Summarized PPF in different time intervals in control (open circles) and wild mice (filled circles). No significant different was detected between two groups.

Paired-pulse facilitation (PPF) is a simple form of synaptic plasticity, and is thought to be related to changes in presynaptic properties. To determine if PPF is affected in slices of wild mice, we performed recordings of PPF in slices from both groups. As shown in Figure 3, we found that PPF was similar between control and wild mice at all intervals tested (35–150 ms) (control group: n = 9 slices from 4 mice; wild group: n = 9 slices from 4 mice; *P *> 0.05; Fig. 3a and 3b).

**Figure 4:**
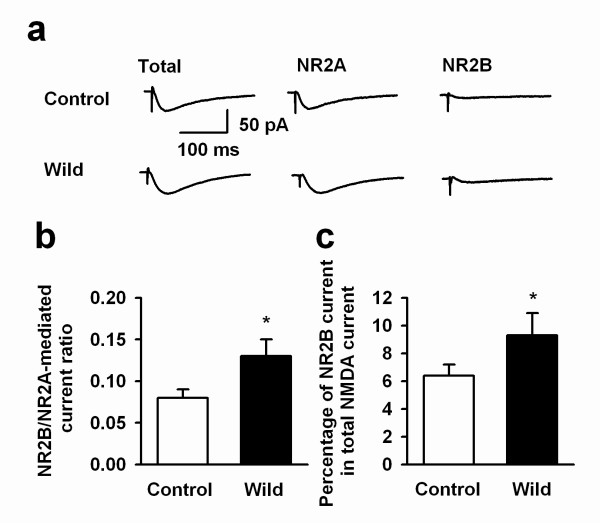
**Enhanced NMDA receptor NR2B/NR2A ratio in the ACC of wild mice**. ***a***: Representative traces showing NMDA receptor NR2A and NR2B subunit-mediated EPSCs from wild and control mice; ***b***: Summary of the NR2B/NR2A ratio in slices of control and wild mice. The NR2B/NR2A current ratio is enhanced in wild mice as compared with that in control mice; **P < 0.01, compared with laboratory mice.

It is well known that NR2B-containing NMDA receptor contributes to synaptic LTP and behavioral learning [11,17,31]. A possible mechanism for an enhancement in ACC LTP is an alteration in NR2B/NR2A subunit composition in slices of wild mice. To test this possibility, we calculated the relative ratios of NR2A- and NR2B-mediated currents after applying selective NMDA NR2A antagonist NVP-AAM077 (0.4 μM) and NR2B antagonist Ro 25–6981 (0.3 μM) to estimate the contribution of NMDA NR2A vs NR2B to the total NMDA receptor mediated EPSC. We found that the ratio NR2B/NR2A mediated EPSCs (i.e., NR2B/NR2A) is significantly higher in wild mice than that in control mice (control group: 0.08 ± 0.01, n = 5 slices from 4 mice; wild group: 0.12 ± 0.03, n = 7 from 4 mice; *t*-test, *P *< 0.05). We also estimated the NMDA NR2B subunit mediated currents over the total NMDA receptor mediated currents; and found that the NR2B/total NMDA EPSCs ratio was significantly greater in wild mice than laboratory mice (control group: 6.4 ± 0.8%, n = 7 slices from 3 mice; wild group: 9.3 ± 1.6%, n = 6 from 3 mice; t-test, P < 0.05; Fig. 4a and 4b). These results suggest that there is more NR2B subunit composition in NMDA receptor-mediated synaptic transmission in the wild mice compared to the control mice within the ACC.

## Discussion

In the present study, we have performed the first study of cingulate plasticity in wild mice. We hypothesize that ACC neuronal synapses are likely modified to fit risky environment as compared with laboratory mice in a comfort animal facility. Our findings demonstrate that natural selection and survival risks facilitate LTP in the ACC. Considering the important contribution of NMDAR NR2B subunits to synaptic LTP and behavioral learning [11,17,31], our results reinforce the role of NMDA NR2B subunit in central plasticity, and more importantly in a 'physiological' condition. Our results also raise the possibility that experimental results from laboratory mice may not the same as those in wild mice. Despite the different in the contribution of NR2B subunit and cingulate LTP, we did find many common features between the neurons of wild mice and that of laboratory mice. Similar basal excitatory responses and PPF were found; suggesting that glutamate mediated excitatory transmission is identical.

The involvement of NMDA NR2B subunit in ACC-related physiological functions and pathological conditions has been reported recently (see [2,27] for reviews). We have demonstrated that inflammation-related pain was selectively increased in mice with forebrain-targeted NR2B overexpression, while physiological or acute pain is not affected [12]. Such genetic manipulation of NMDA NR2B subunit overexpression can be at least mimicked in pathological conditions. For example, peripheral inflammation at one hindpaw in adult mice increased the expression level of NR2B proteins in the ACC [17]. Moreover, we have demonstrated that the exposure to enriched environment enhanced behavioral nociceptive responses to peripheral inflammation [10]. Consistently, we have shown that the NMDA NR2B receptors are involved in synaptic plasticity and contextual fear memory [13,14]. Together with the present findings, we argue that NMDA NR2B subunit is highly sensitive to an enriched environment and peripheral injury. Future studies are clearly needed to investigate the molecular mechanism for the upregulation of NMDA NR2B subunit in central neurons.

Considering the increases in NMDAR subunit NR2B/NR2A current ratio in the wild mice, we expect that wild mice are likely superior in learning and memory as compared with control mice. Dissecting the molecular and cellular mechanisms in cingulate synaptic plasticity by using the wild mice may help us to understand the new insights of cortical plasticity and its related physiological functions.

## Methods

### Animals

We used adult wild mice captured by the Mouse/insect glue traps (plastic board). Usually, experiments were performed 4–8 hours after the mice were caught. The body weight of wild mice ranged from 22.5–31 gm. For comparison, eight-week-old C57BL/6 male mice with similar body weight were used as the laboratory control mice. These control mice were housed under a 12:12 light cycle with food and water provided *ad libitum*. The Animal Care and Use Committee of the Fourth Military Medical University (China) approved the animal protocol.

Brain slice electrophysiologyCoronal brain slices (300 μm) from wild mice or laboratory mice, containing the ACC, were prepared using standard methods as previously described [12]. Slices were transferred to a submerged recovery chamber containing oxygenated (95% O_2 _and 5% CO_2_) artificial cerebrospinal fluid (ACSF) (in mM: 124 NaCl, 2.5 KCl, 2 CaCl_2_, 1 MgSO_4_, 25 NaHCO_3_, 1 NaH_2_PO_4_, 10 glucose) at room temperature for at least 1 h.

Experiments were performed in a recording chamber on the stage of an Axioskop 2FS microscope with infrared DIC optics for visualizing whole-cell patch clamp recordings. Excitatory postsynaptic currents (EPSCs) were recorded from layer II-III neurons using an Axon 200B amplifier (Axon Instruments, CA) and stimulations were delivered using a bipolar tungsten stimulating electrode placed in layer V of the ACC. The α-amino-3-hydroxy-5-methyl-4-isoxazolepropionic acid (AMPA) receptor-mediated EPSCs were induced by repetitive stimulations at 0.02 Hz and neurons were voltage clamped at -70 mV. The recording pipettes (3–5 MΩ) were filled with solution containing (mM) 145 K-gluconate, 5 NaCl, 1 MgCl_2_, 0.2 EGTA, 10 HEPES, 2 Mg-ATP, and 0.1 Na_3_-GTP (adjusted to pH 7.2 with KOH).

LTP was induced within 12 min after obtaining stable EPSCs to prevent the washout effect. The protocol involved paired presynaptic 80 pulses at 2 Hz with postsynaptic depolarization at +30 mV (referred to as pairing training). The NMDA receptor-mediated component of EPSCs was pharmacologically isolated in ACSF containing: CNQX (20 μM) and picrotoxin (100 μM). The patch electrodes contained (in mM) 102 cesium gluconate, 5 TEA-chloride, 3.7 NaCl, 0.2 EGTA, 20 HEPES, 2 MgATP, 0.3 NaGTP, and 5 QX-314 chloride (adjusted to pH 7.2 with CsOH). Neurons were voltage clamped at -30 mV and NMDA receptor-mediated EPSCs were evoked at 0.05 Hz. NVP-AAM077 (0.4 μM) and Ro25–6981 (0.3 μM) were bath applied sequentially to assess the NR2A- and NR2B-components of EPSCs. For AMPA EPSC/NMDA EPSC ratio experiments, AMPA receptor mediated EPSCs were recorded at a holding potential of -70 mV and picrotoxin (100 μM) was added to ACSF to inhibit IPSCs. After recording AMPA receptor-mediated currents, NBQX (10 μM) was added to ACSF and holding potential was changed to +40 mV to record NMDA receptor mediated EPSCs. The AMPA/NMDA ratio was calculated by the peak amplitude of mean traces. The access resistance was 15–30 MΩ and was monitored throughout the experiment. Data were discarded if access resistance changed by more than 15% during an experiment.

### Data analyses

Results were analyzed by t-test, paired t-test, or two-way ANOVA followed by post-hoc Student-Newman-Keuls test to identify significant differences. All data are expressed as mean ± S.E.M. In all cases, *P *< 0.05 was considered statistically significant.

## Abbreviations

ACC: anterior cingulate cortex; ACSF: artificial cerebrospinal fluid; AMPA: α-amino-3-hydroxy-5-methyl-4-isoxazolepropionic acid; EE: enriched environmental; EPSC: excitatory postsynaptic current; LTP: long-term potentiation; NMDA: N-methyl D-aspartate receptor; PPF: paired-pulse facilitation.

## Competing interests

The authors declare that they have no competing interests.

## Authors' contributions

MGZ is responsible for performance of electrophysiology and writing the manuscript. HT is responsible for performance of electrophysiology. YKW is responsible for some experiments as well as data analyses. MZ is responsible for experimental design and writing the manuscript. All authors read and approved the final manuscript.
